# Quantitative response assessment of combined immunotherapy in a murine melanoma model using multiparametric MRI

**DOI:** 10.1186/s41747-025-00597-8

**Published:** 2025-06-14

**Authors:** Maurice M. Heimer, Amra Cimic, Sandra Kloiber-Langhorst, Melissa J. Antons, Jennifer Stueckl, Heidrun Hirner-Eppeneder, Wolfgang G. Kunz, Olaf Dietrich, Jens Ricke, Felix L. Herr, Clemens C. Cyran

**Affiliations:** Department of Radiology, LMU University Hospital, LMU Munich, Munich, Germany

**Keywords:** Immunotherapy, Melanoma, Mice (inbred C57BL), Multiparametric magnetic resonance imaging, Tumor microenvironment

## Abstract

**Background:**

We assessed immunotherapy response in a murine melanoma model using multiparametric magnetic resonance imaging (mpMRI) features with *ex vivo* immunohistochemical validation.

**Methods:**

Murine melanoma cells (B16-F10) were inoculated into the subcutaneous flank of *n* = 28 C57BL/6 mice (*n* = 14 therapy; *n* = 14 control). Baseline mpMRI was acquired on day 7 at 3 T. The immunotherapy group received three intraperitoneal injections of anti-PD-L1 and anti-CTLA-4 antibodies on days 7, 9, and 11 after inoculation. Controls received a volume equivalent placebo. Follow-up mpMRI was performed on day 12. We assessed tumor volume, diffusion-weighted imaging parameters, including the apparent diffusion coefficient (ADC), and dynamic-contrast-enhanced metrics, including plasma volume and plasma flow. Tumor-infiltrating lymphocytes (TIL; CD8+), cell proliferation (Ki-67), apoptosis (terminal deoxynucleotidyl transferase deoxyuridine triphosphate nick-end labeling, TUNEL), and microvascular density (CD31+) were assessed in a validation cohort of *n* = 24 animals for time-matched *ex vivo* validation.

**Results:**

An increase in tumor volume was observed in both groups (*p* ≤ 0.004) without difference at follow-up (*p* = 0.630). A lower ADC value was observed in the immunotherapy group at follow-up (*p* = 0.001). Immunohistochemistry revealed higher TUNEL values (*p* < 0.001) and CD8+ TILs (*p* = 0.048) following immunotherapy, as well as lower tumor cell Ki-67 values (*p* < 0.001) and microvascular density/CD31+ (*p* < 0.001).

**Conclusion:**

Lower tumor ADC, paired with higher intratumoral expression of CD8+ TIL, was observed five days after immunotherapy, suggestive of early immunological response. *Ex vivo* immunohistochemistry confirmed the antitumoral efficacy of immunotherapy.

**Relevance statement:**

Compared to tumor size, diffusion-weighted MRI demonstrated potential for early response assessment to immunotherapy in a murine melanoma model, which could reflect changes in the tumor microenvironment and immune cell infiltration.

**Key Points:**

- No difference in tumor volume was observed between groups before and after therapy.
- Lower ADC values paired with increased CD8+ TILs were observed following immunotherapy.
- *Ex vivo* immunohistochemistry confirmed antitumoral efficacy of anti-PD-L1 and anti-CTLA-4 immunotherapy.

**Graphical Abstract:**

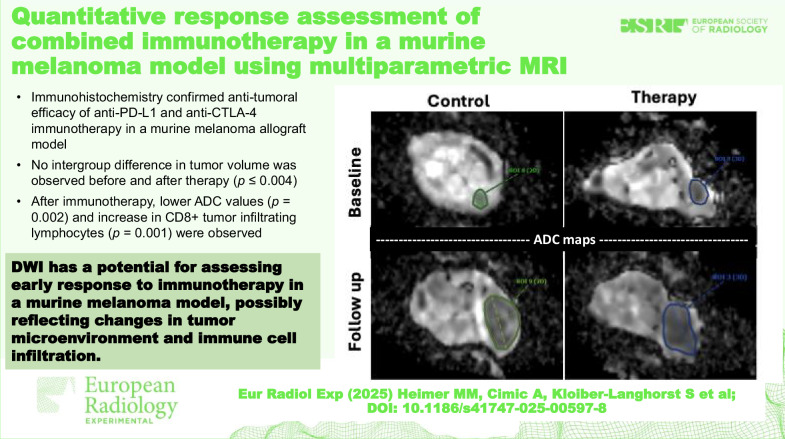

## Background

As immunotherapy continues to expand as a pivotal therapeutic option for melanoma and a wide range of cancers, precise detection and comprehensive assessment of tumor responses are essential for advancing efficacy and personalization of cancer immunotherapy [1–3]. Significant challenges remain, particularly in identifying predictive biomarkers for patient stratification before therapy and optimizing response assessment methods [4, 5]. Checkpoint inhibitors, such as PD-1 and CTLA-4 blockers, function by preventing interactions between PD-1 and PD-L1 on tumor cells or between CTLA-4 and CD80+ on antigen-presenting cells [2, 6–9]. This mechanism enhances T cell activity and leads to increased cytotoxic T cell infiltration into tumors, resulting in transient tumor enlargement, followed by tumor shrinkage or long-term stability [10, 11]. However, this transient increase in tumor volume may be misinterpreted as disease progression under the standard response evaluation criteria in solid tumors (RECIST v1.1), which defines tumor growth as disease progression [12].

This phenomenon, known as pseudoprogression, has been documented in numerous studies and has been reported to reach up to 6–10% in melanoma patients and appears as a relevant challenge in early response assessment [5, 12, 13]. Modified criteria, including iRECIST, have aimed to address this phenomenon, however require additional follow-up imaging and fail to assess early response [4, 14, 15]. Beyond morphology, immune positron emission tomography response criteria in solid tumors (iPERCIST) derived from [^18^F]-FDG positron emission tomography/computed tomography have shown value in immunotherapy response assessment but may also require further validation [16, 17].

Currently, no routine clinical imaging biomarkers are available to reliably distinguish between immunotherapy-associated pseudoprogression and true progressive disease during the early period after immunotherapy. The presence of tumor-infiltrating lymphocytes (TILs) has emerged as a potential biomarker for predicting response to PD-1 blockade, particularly in metastatic melanoma [3, 18–20]. However, detecting TILs typically requires invasive biopsies, which are hindered by sampling errors, tumor localization, and the static nature of *ex vivo* assessments [21, 22].

To address these limitations, non-invasive, dynamic imaging biomarkers are gaining traction for their ability to monitor therapy responses longitudinally and assess intratumoral and metastatic heterogeneity [23, 24]. Multiparametric magnetic resonance imaging (mpMRI) has evolved as a versatile, widely available modality offering a wealth of quantitative data by integrating high-resolution morphological imaging with functional techniques including diffusion-weighted imaging (DWI) and dynamic contrast-enhanced MRI (DCE-MRI) studies [25]. These quantitative imaging biomarkers hold the potential to non-invasively assess the molecular and cellular characteristics of the tumor microenvironment *in vivo*, including its spatial and temporal heterogeneity, ultimately offering a powerful tool for monitoring immunotherapy response [26–29].

We hypothesized that quantitative mpMRI parameters could non-invasively monitor immunotherapy and potentially identify intratumoral immune infiltration. In this study, we aimed to investigate quantitative mpMRI biomarkers in reference to *ex vivo* immunohistochemistry to assess early immunotherapy response in a murine melanoma model.

## Methods

### Animal model and experimental protocol

All experiments were approved by local authorities (ROB-55.2-2532.Vet_02-19-32) and conducted in compliance with guidelines for the use of living animals in scientific research. Female, 10–12-week-old C57BL/6 mice (Charles River) were used in this experiment and housed under species-specific husbandry conditions. After a one-week acclimation period, B16-F10 cells (3 × 10^5^, ATCC CRL-6475) were resuspended in a 1:1 mixture of Matrigel (BD Biosciences, San Jose, CA, USA) and phosphate-buffered saline (pH 7.4) and injected subcutaneously into the left dorsal flank of C57BL/6 mice (*n* = 28) under inhalative isoflurane anesthesia (2.0 vol% isoflurane, 1.5 L/min oxygen). Animals were randomly assigned to the therapy group (*n* = 14) or control group (*n* = 14). The therapy group received three intraperitoneal injections of anti-PD-L1 and anti-CTLA-4 antibodies (20 µg/kg) on days 7, 9, and 11 after tumor inoculation, following baseline imaging. The control group received a volume-equivalent intraperitoneal placebo. MRI was performed 7 days (baseline) and 12 days (follow-up) after inoculation. In compliance with regulations made by the local authority, exclusion criteria were defined a priori, including ≥ 20% weight loss, tumor growth beyond 1.5 cm in longest diameter, tumor exulceration, infections or bleeding from the tumor area, bloody diarrhea, apathy, ascites, or acneiform dermatitis.

### MRI protocol

MRI was performed on a clinical 3-T scanner (MAGNETOM Skyra, Siemens Healthineers, Erlangen, Germany). Under isoflurane anesthesia (2.0 vol% isoflurane, 1.0 L/min oxygen), animals were placed in a dedicated animal bed and a 16-channel wrist coil (Siemens Healthineers, Erlangen, Germany) in the head-first prone position. The animal bed was connected to a warming system to maintain animal body temperature of 36 ± 0.5 °C, which was monitored by a rectal probe. Breathing rate was monitored by a respiratory bellows. Sequences were acquired without gating techniques. The detailed protocol is displayed in the Supplemental Materials. DCE-MRI sequences were acquired following weight-adjusted (4 mmol/kg bodyweight) intravenous administration of saline-diluted (1:25) gadobutrol (0.1 mmol/mL, Gadovist, Bayer, Berlin, Germany) using a tail vein catheter access. The contrast agent was injected using a dedicated injection system (PHD2000 Series, Harvard Apparatus, Holliston, MA, USA), ensuring equivalent injection rates between scans and animals.

### MRI data processing

Images were analyzed and segmented using a clinical Picture Archiving and Communication System workstation (Visage Imaging, San Diego, CA, USA). Volumetric handcrafted regions of interest were placed individually in T1-weighted morphological, DWI- and DCE-MRI data in a consensus reading of two readers (M.M.H. and A.C.) with 8 years and 1 year of experience in preclinical imaging, respectively. For DCE-MRI, an additional volumetric region of interest was placed in the abdominal aorta to generate an arterial input function. Signal intensity *versus* time curves were extracted using whole tumor volumes of interest and the arterial input function. No measurements were reported when tumors were too small for reliable segmentation and assessment.

All DWI data were in-plane motion corrected and smoothed with a volumetric Gaussian filter (width 1.5 mm); data with signal dropout due to motion were excluded. Signal drop out was defined as a mean foreground signal intensity of an individual repetition being lower than 2/3 = 67% of the mean foreground signal intensity of the brightest 50% of all image repetitions of the same slice and *b*-value. For DWI, apparent diffusion coefficient (ADC) values (in mm²/s) were calculated voxel-wise using nonlinear fitting to a monoexponential signal model. Also, a non-monexponential intravoxel incoherent motion (IVIM) model was evaluated; data was fitted voxelwise using a triexponential model that included perfusion-related (microcapillary) pseudodiffusion (D*) in mm²/s and perfusion fraction (*f*) in %, free water diffusion *D*_water_ = 3.0 × 10^-3^ mm^2^/s (fixed), fitted water fraction *f*_water_ in %, and tissue diffusion (*D*) in mm²/s. DCE-MRI data were three-dimensionally motion corrected and smoothed with a volumetric Gaussian filter (width 1.0 mm). The (absolute) signal enhancement *S*(*t*) − *S*_0_ (*S*(*t*) is the signal intensity; *S*_0_ is signal intensity prior to arrival of the contrast agent) was used to approximate the contrast-agent concentration. Then tracer-kinetic modeling with a two-compartment exchange model was performed to determine voxel-wise the plasma flow (PF) in mL/100 mL/min and the plasma volume (PV) in mL/100 mL. Mean values were used for further analysis.

### Immunohistochemistry

A total of *n* = 24 C57BL/6 mice were dedicated to *ex vivo* immunohistochemistry following the aforementioned experimental protocol. Seven days after tumor inoculation, *n* = 8 animals were sacrificed for baseline imaging time point validation. Tumors were explanted, formalin-fixed, and cryopreserved for multiparametric *ex vivo* immunohistochemical analysis, including CD8+ cells (TILs), tumor cell proliferation (Ki-67), apoptosis (TUNEL, see below), and microvascular density (CD31+). The remaining cohort was randomized into *ex vivo* validation therapy (*n* = 8) and control group (*n* = 8), receiving treatment as described above. Animals of this cohort were sacrificed 12 days after inoculation for *ex vivo* tumor analysis.

#### CD31

Deparaffinization, rehydration, and antigen retrieval were performed using EDTA-Unmasking Solution (SignalStain®, Cell Signaling Technology, Danvers, MA, USA). For immunohistochemical assessment of tumor microvascular density, non-specific binding sites were blocked with 5% donkey serum in TBS-Tween 20. Tumor slices were incubated overnight with a polyclonal rabbit anti-CD31 primary antibody (1:50; Abcam ab28364, Abcam Limited, Cambridge, UK). Secondary antibodies conjugated with Alexa Fluor 488 (1:200; Abcam ab150073, Abcam Limited, Cambridge, UK) were applied for fluorescence detection. Counterstaining was performed with 4′,6-diamidino-2-phenylindole (DAPI) (Carl Roth, Karlsruhe, Germany), and slides were mounted with Fluoromount-G (Thermo Fisher Scientific, Waltham, MA, USA). Tumor microvessels were quantified as the mean number of endothelial cells in ten random fields at 200× magnification.

#### CD8 antigen staining

Tissue slides were deparaffinized and rehydrated, followed by permeabilization with 1× phosphate-buffered saline (Gibco, Waltham, MA, USA) and 0.25% Triton-X-100 (Merck KGaA, Darmstadt, Germany). Antigen retrieval was performed by boiling slides in 10 mM Tris-EDTA buffer (Thermo Fisher Scientific, Waltham, MA, USA) for CD8. After antigen retrieval, slides were washed with 1× phosphate-buffered saline to remove residual buffers. Nonspecific binding was blocked using bovine serum albumin, and primary antibodies against CD8 (1:50; Abcam ab217344, Abcam Limited, Cambridge, UK) were applied and incubated overnight. Following additional washes, secondary antibodies conjugated with Alexa Fluor 488 (1:200; Abcam ab150073, Abcam Limited, Cambridge, UK) were applied for fluorescence detection. Counterstaining with DAPI enabled visualization of nuclei. Slides were mounted with Fluoromount-G (Thermo Fisher Scientific, Waltham, MA, USA) for optimal preservation and visualization. CD8+ T-cells were quantified as the mean percentage in ten randomly selected fields at 200× magnification.

#### Ki-67 antigen staining

A Ki-67-specific monoclonal rabbit antihuman antibody (1:50; SP6, Thermo Fisher MA5-14420, Thermo Fisher Scientific, Waltham, MA, USA) was used to quantify tumor cell proliferation. Antigen retrieval was performed with Universal Antigen Retrieval Reagent (ab208572, Abcam Limited, Cambridge, UK) using microwave irradiation at 600 W. After washing with distilled water and TBS-Tween (0.05%), secondary antibodies conjugated with Alexa Fluor 488 (1:200; ab150073, Abcam Limited, Cambridge, UK) were applied for fluorescence detection. Counterstaining was carried out with DAPI (Carl Roth, Karlsruhe, Germany), and slides were mounted with Fluoromount-G (Thermo Fisher Scientific, Waltham, MA, USA). Proliferating cells were quantified as the mean percentage in ten randomly selected fields at 200× magnification.

#### Terminal deoxynucleotidyl transferase deoxyuridine triphosphate (dUTP) nick-end labeling (TUNEL)

Tissue slides were deparaffinized and rehydrated, followed by permeabilization with a solution containing Triton X-100 (Merck KGaA, Darmstadt, Germany) and sodium citrate (Sigma-Aldrich, St. Louis, MO, USA). Antigen retrieval was performed using antigen retrieval reagent (ab208572, Abcam Limited, Cambridge, UK) with microwave treatment. Staining was carried out using a kit, ensuring temperature control, and maintaining darkness during the process. After thorough washing, slides were counterstained with DAPI to visualize nuclei. Finally, slides were mounted with Fluoromount-G (Thermo Fisher Scientific, Waltham, MA, USA) to preserve the stained specimens for microscopic examination. Apoptotic cells were quantified as the mean percentage in ten randomly selected fields at 200× magnification.

### Statistical analysis

Continuous variables are presented as mean ± standard deviation. Nonparametric testing was used for statistical evaluation, due to small cohort sizes. Intergroup comparisons between control and therapy groups were conducted for baseline and follow-up using the Mann–Whitney *U*-test. The Wilcoxon test was used for intragroup comparison after pairwise deletion for missing values to ensure coherence between assessments. Also, correlation was assessed for quantitative image parameters using Spearman correlation testing. All statistical analyses and visualization were performed using R, version 4.3.0 (R Core Team 2025). Statistical significance was considered for *p*-values < 0.050. Due to the exploratory study design, significance levels were not adjusted for multiple comparisons.

## Results

### Protocol completion

Imaging was completed in *n* = 27 animals of the experimental cohort; one animal in the control group was terminated before study completion due to previously defined tumor volume-based exclusion criteria.

### Tumor volume

**Fig. 1:**
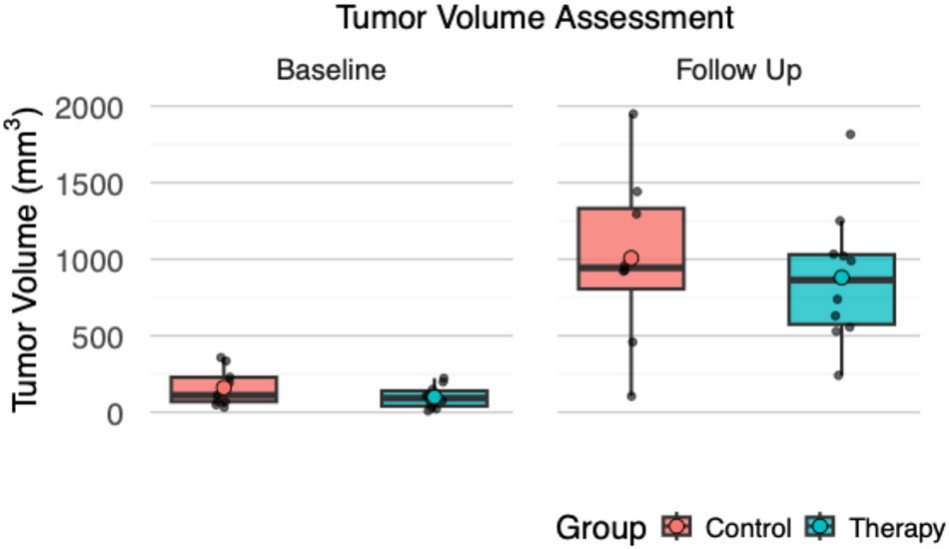
Tumor volume assessment. Both control and therapy groups displayed a significant increase in tumor volume at follow-up, five days after baseline imaging (*p* ≤ 0.004). No significant intergroup difference was observed at follow-up (*p* = 0.630)

**Fig. 2:**
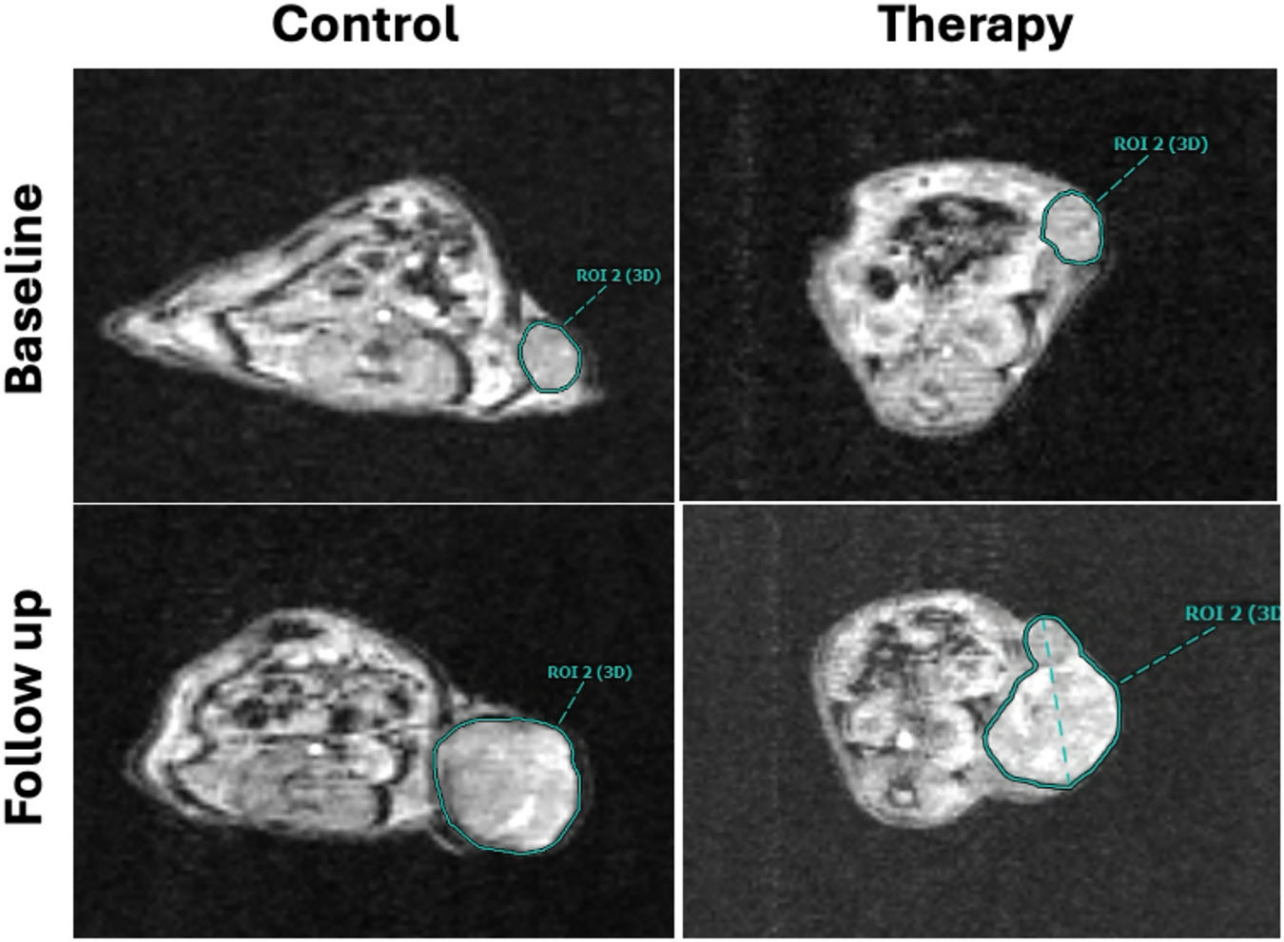
Representative axial T1-weighted image for tumor volume assessment: Significant tumor growth was observed between baseline and follow-up in both control (71 mm^3^ to 1,033 mm^3^) and therapy group (107 mm^3^ to 1,033 mm^3^) in the displayed images. No significant difference was observed between groups

At baseline, *n* = 12 tumors of the therapy group and *n* = 9 tumors of the control group were assessed, the remaining tumors were too small for assessment. Tumor volume did not significantly differ between control and therapy group at baseline (160 mm^3^ ± 117 mm^3^ *versus* 121 mm^3^ ± 84 mm^3^; *p* = 0.508) or follow-up (1,009 mm^3^ ± 715 mm^3^ *versus* 781 mm^3^ ± 447 mm^3^; *p* = 0.630; Fig. 1). Observed tumor growth was significant in both the control (*p* = 0.004) and therapy group (*p* = 0.002). Representative T1-weighted images are shown in Fig. 2.

### Diffusion-weighted MRI

#### Monoexponential ADC model

**Fig. 3:**
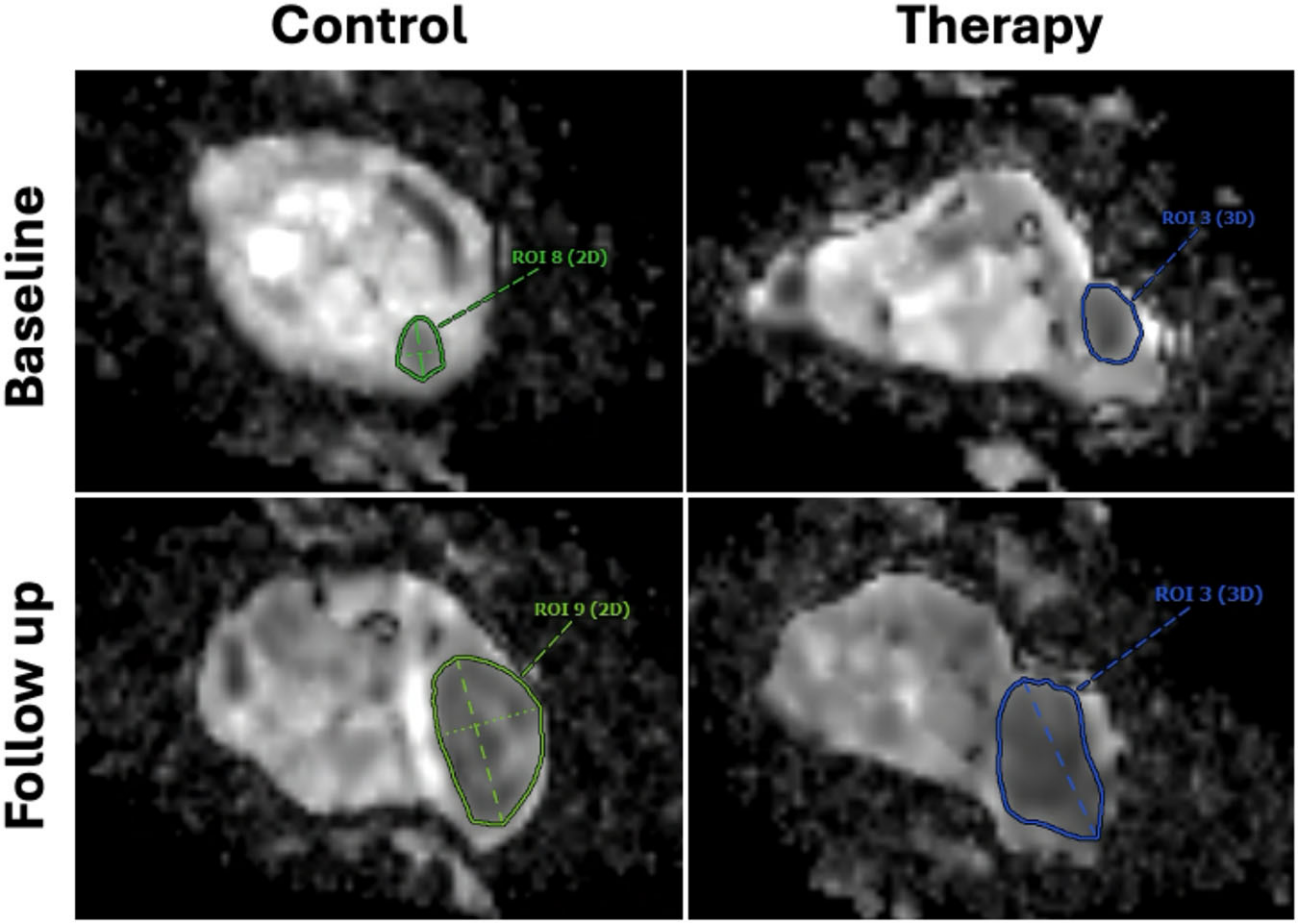
Representative monoexponential ADC maps: Note there was a significantly lower ADC in the immunotherapy group compared to the control group at follow-up (0.759 × 10^-3^ mm^2^/s *versus* 0.808 × 10^-3^ mm^2^/s) in displayed images, no difference was observed at baseline (0.727 × 10^-3^ mm^2^/s *versus* 0.723 × 10^-^^3^ mm^2^/s). ADC, Apparent diffusion coefficient

**Fig. 4:**
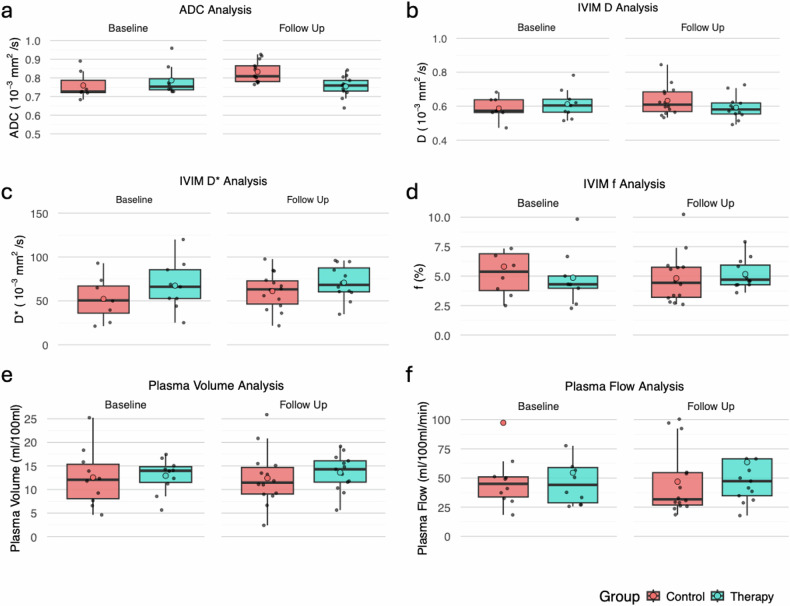
mpMRI assessment: Note the significant differences in **a** demonstrating a significantly lower ADC at follow-up compared to baseline in the control group (*p* = 0.002) and in **c** showing a significantly higher D* at follow-up compared to baseline in the control group (*p* = 0.008). All other intra- and intergroup differences in IVIM MRI (**b**, **d**) and DCE-MRI (**e**, **f**) were not significant (all *p* ≥ 0.139). ADC, Apparent diffusion coefficient; *D*, Free water diffusion; D*, Pseudodiffusion; DCE, dynamic contrast enhanced (MRI); *f*, Perfusion fraction; IVIM, Intravoxel incoherent motion; MRI, Magnetic resonance imaging

At baseline, the tumor was detected in *n* = 7 animals in the control group and in *n* = 8 animals in the therapy group, with no significant intergroup difference in ADC (0.759 ± 0.069 × 10^-3^ mm^2^/s *versus* 0.786 ± 0.077 × 10^-^^3^ mm^2^/s; *p* = 0.189). At follow-up, a significantly higher ADC was observed in the control group compared to the therapy group (0.833 ± 0.054 × 10^-^^3^ mm^2^/s *versus* 0.754 ± 0.051 × 10^-^^3^ mm^2^/s; *p* = 0.001). In both control (0.759 ± 0.069 × 10^-3^ mm^2^/s *versus* 0.814 ± 0.067 × 10^-^^3^ mm^2^/s; *p* = 0.297) and immunotherapy group (0.759 ± 0.069 × 10^-^^3^ mm^2^/s *versus* 0.814 ± 0.044 ×10^-^^3^ mm^2^/s; *p* = 0.578), no significant differences were observed between baseline and follow-up. Representative ADC maps are shown in Fig. 3, data are shown in Fig. 4a.

#### Non-monexponential IVIM model

At baseline the tumor was detected in *n* = 8 animals in the control group and *n* = 9 animals in the therapy group, with no significant intergroup difference of *D* (0.588 ± 0.060 ×10^-^^3^ mm^2^/s *versus* 0.613 ± 0.080 × 10^-^^3^ mm^2^/s; *p* = 0.673), D* (52.3 ± 22.7 × 10^-^^3^ mm^2^/s *versus* 67.4 ± 26.7 × 10^-^^3^ mm^2^/s; *p* = 0.139) and *f* (5.81 ± 2.77% *versus* 4.86 ± 2.14%; *p* = 0.541). Also, no significant differences were observed at follow-up between control and therapy group with regard to *D* (0.633 ±0.084 × 10^-^^3^ mm^2^/s *versus* 0.592 ± 0.067 × 10^-^^3^ mm^2^/s; *p* =0.297), D* (61.0 ± 20.2 × 10^-^^3^ mm^2^/s *versus* 70.9 ± 18.6 ×10^-^^3^ mm^2^/s; *p* = 0.322) and *f* (4.82 ± 2.07% *versus* 5.17 ±1.20%; *p* = 0.321). A significant intragroup increase in D* was seen in the control group (52.3 ± 22.7 × 10^-^^3^ mm^2^/s *versus* 75.5 ± 15.5 × 10^-^^3^ mm^2^/s; *p* = 0.008), but not in the therapy group (*p* = 0.078). Data are shown in Fig. 4b, d.

### DCE-MRI

At baseline the tumor was detected *n* = 8 animals in the control group and in *n* = 9 animals in the therapy group in DCE-MRI, with no significant intergroup difference of PF (97.4 ± 167.9 mL/100 mL/min *versus* 54.5 ± 35.6 mL/100 mL/min; *p* = 0.971) and PV (12.6 ± 5.8 mL/100 mL *versus* 12.9 ± 3.4 mL/100 mL; *p* = 0.280). Also, no significant differences were observed at follow-up between control and therapy group for PF (46.9 ± 28.0 mL/100 mL/min *versus* 63.6 ± 52.2 mL/100 mL/min; *p* = 0.631) and PV (12.4 ± 5.6 mL/100 mL *versus* 13.6 ± 3.7 mL/100 mL; *p* = 0.280), with data shown in Fig. 4e, f.

### Correlation analysis

**Fig. 5:**
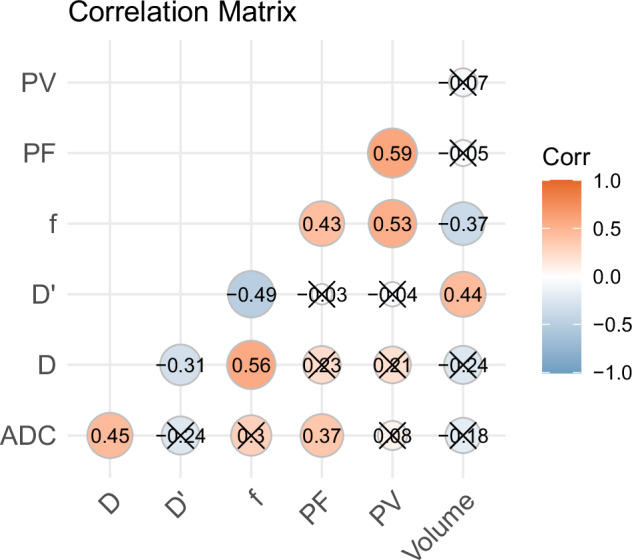
Assessment of quantitative MRI parameters in a correlation matrix: The correlation coefficient (ρ) is displayed in the individual data points, with a data legend on the right. The magnitude of correlation is proportional to the circle size and color intensity. Nonsignificant correlation (*p* ≥ 0.05) is indicated by a cross overlaying the data point. ADC, Apparent diffusion coefficient; *D*, Free water diffusion; D*, Pseudodiffusion; DCE, Dynamic contrast enhanced (MRI): *f*, Perfusion fraction; IVIM, intravoxel incoherent motion; MRI, Magnetic resonance imaging; PF, Plasma flow; PV, Plasma volume

A significant moderate correlation was found between the parameters PF and PV (ρ = 0.59; *p* < 0.001), *D* and *f* (ρ = 0.56; *p* < 0.001), as well as PV and *f* (ρ = 0.53; *p* < 0.001). A weak correlation was found between D* and *f* (ρ = -0.49; *p* < 0.001), ADC and *D* (ρ = 0.45; *p* = 0.014), D* and *D* (ρ = -0.31; *p* = 0.041), ADC and PF (ρ = 0.37; *p* = 0.032), PF and *f* (ρ = 0.43; *p* = 0.004), *f* and tumor volume (ρ = -0.37; *p* = 0.016), as well as D* and tumor volume (ρ = 0.44; *p* = 0.003). All other values displayed no significant correlation as demonstrated in Fig. 5.

### Immunohistochemistry

**Fig. 6:**
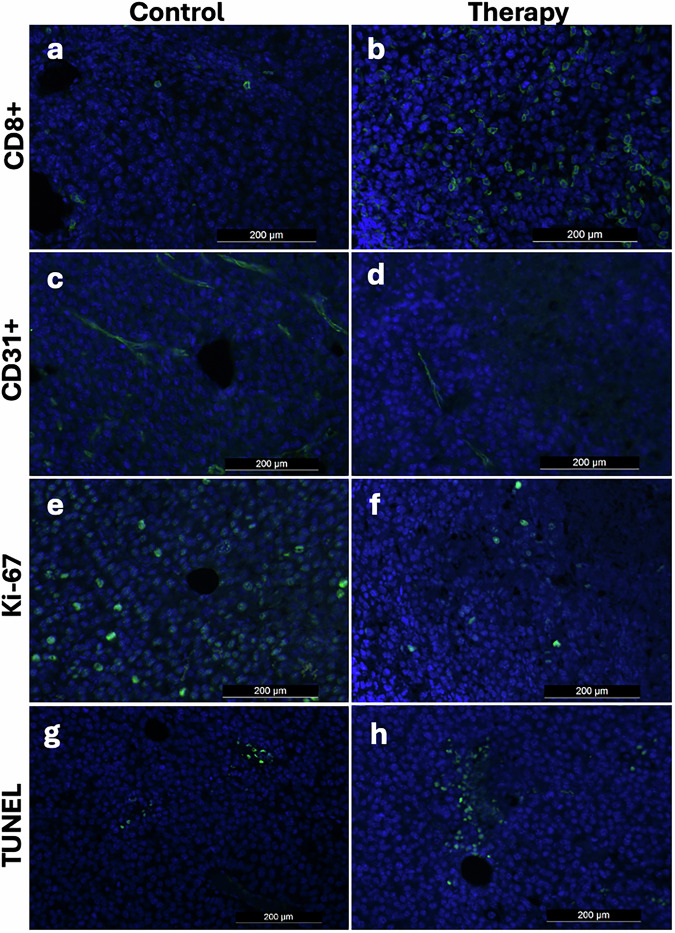
Representative fluorescence immunohistochemistry images. Results from the control group are displayed in the left column, from the therapy group on the right; CD8+ (tumor infiltrating lymphocytes) (**a,** **b**), CD31+ (microvascular density) (**c,** **d**), Ki-67 (cell proliferation) (**e**, **f**), TUNEL (apoptosis) (**g**, **h**). Note that the therapy group exhibited a significantly higher tumor immune infiltration and apoptosis rate, as well as a significantly lower microvascular density and tumor cell proliferation. TUNEL, Terminal deoxynucleotidyl transferase deoxyuridine triphosphate (dUTP) nick-end labeling

The *ex vivo* analysis revealed significant differences between the control and therapy group at follow-up; tumors in the therapy group had significantly higher percentage of apoptotic cells (TUNEL: 17.1 ± 5.6% *versus* 52.3 ± 17.2%; *p* = 0.001), higher number of TILs (CD8+: 71.9 ± 55.6 *versus* 395.4 ± 522.5; *p* = 0.049) and significantly lower tumor cell proliferation (Ki-67: 58.7 ± 8.6% *versus* 27.2 ± 9.7%; *p* = 0.001) than the control group. In addition, significantly lower microvascular density was observed following immunotherapy at follow-up (CD31+: 286.3 ± 88.1 *versus* 142.1 ± 59.8; *p* = 0.003) compared to the control group. Following immunotherapy, a significant increase of apoptotic cells (TUNEL: 16.2 ± 6.9 *versus* 52.3 ± 17.2; *p* < 0.001), and TIL (CD8+: 28.4 ± 30.7 *versus* 395.4 ± 522.5; *p* = 0.001) was observed between baseline and follow-up, as well as significantly lower microvascular density (CD31+: 304.7 ± 173.7 *versus* 142.1 ± 59.8; *p* = 0.030) and reduced tumor cell proliferation (Ki-67: 56.1 ± 14.6 *versus* 27.2 ± 9.7; *p* = 0.002). The control group displayed no significant changes in any of the immunohistochemical parameters (all *p* ≥ 0.056). Representative images are shown in Fig. 6.

## Discussion

This study evaluated the potential of clinically available quantitative mpMRI biomarkers for early, non-invasive response assessment following a combined intraperitoneal immunotherapy in a murine melanoma model. Five days after therapy, the immunotherapy group demonstrated significantly lower ADC values paired with a significant increase in CD8+ lymphocytes compared to the control group, reflecting therapeutic immune infiltration of tumors. *Ex vivo* validation confirmed significant antitumoral response on immunotherapy after five days of treatment, characterized by anti-proliferative, anti-angiogenic, pro-apoptotic effects, as well as pronounced lymphocytic immune infiltration into the tumor.

These findings align with prior studies demonstrating early therapeutic responses in murine models, particularly the infiltration of CD8+ T cells, a key determinant of immunotherapy success [30–34]. In a preclinical study of colorectal adenocarcinoma, Jiang et al identified early temporal changes in the mean cellular size of the tumor microenvironment in a small animal model after immunotherapy [30]. However, in contrast to our study, they found a significant decrease in ADC of the control group as early as six days after therapy, but not between groups [30]. This is in line with findings by Fliedner et al, who have reported ADC as non-invasive, tissue cellularity marker following radiotherapy [35]. Also, a decline in ADC was observed following a seven-day treatment with dabrafenib and ribociclib in an A375 experimental murine melanoma, which may reflect different therapeutic mechanisms [36]. Multiple cellular pathways and therapeutic effects may coexist, contributing to differences among tumor entities. Tumor immune infiltration, characterized by the recruitment of immune cells to the tumor site, may result in a transient increase in cellular density within the tumor microenvironment [28]. A previous preclinical study demonstrated that cytotoxic therapy-induced necrosis, compared to apoptosis alone, was associated with a more pronounced increase in ADC [37]. Also, marked tumor immune infiltration may lead to significantly reduced extracellular volume and therefore ADC during the initial phase of T cell migration [25]. Therefore, an increase in TIL and overall tumor cellularity following immunotherapy may mask or counteract potential increases in ADC that would otherwise result from tumor necrosis or apoptosis. Further research is warranted to investigate this observation.

The IVIM model has emerged as an advanced multiparametric DWI technique that enables the differentiation of diffusion and perfusion components at the microvascular level, accounting for capillary blood flow [38, 39]; the true diffusion coefficient (*D*) reflects the diffusion of water molecules and is associated with cellular density, whereas the pseudo-diffusion coefficient D* represents microcirculation perfusion, with *f* denoting the perfusion fraction of flowing blood [39].

While the first studies investigating IVIM demonstrated promising results in oncology imaging [16, 39–42], evidence for applications in melanoma and immunotherapy assessment remains limited. While *D*, D*, and *f* failed to distinguish between control and therapy groups, a significant increase in D* was observed in the control group between baseline and follow-up, which, however, only showed a weak correlation to *D* and *f*, but not to ADC or DCE perfusion metrics. Our study also demonstrated a weak correlation between ADC and *D*, which is not surprising as *D* is the predominant quantity determining the ADC. In a preclinical glioblastoma study, Hu et al reported a strong correlation between D* and CD31+ expression, as well as *D* and TUNEL staining following cyclophosphamide therapy, which may suggest their value as imaging biomarkers [37]. Similarly, a preclinical study on colon adenocarcinoma demonstrated a moderate correlation between D* and *f* to immunohistochemistry parameters of tumor angiogenesis and *D* demonstrating strong correlation to tumor cell proliferation, apoptosis and infiltration of lymphocytes, with significant differences as early as 6 days after therapy, a time point not assessed in our study [31].

Similarly, PV and PF assessed by DCE-MRI did not yield significant results to help differentiate control and therapy groups in our study, with moderate correlation between the two parameters, a moderate correlation between PV and *f*, as well as a weak correlation between PF and *f*, as well as ADC, as shown before [31]. While DCE-MRI provides insights into tumor vascularity and perfusion, and the IVIM model assesses microvascular perfusion and diffusion characteristics, both methods may lack the sensitivity to detect the nuanced cellular and molecular changes induced by immunotherapy in this early interval. Previous studies demonstrated that early response assessment is feasible seven days after treatment following non-immunotherapy treatment of colorectal and prostate cancer [43, 44]. While mpMRI has not yet been fully validated in large-scale human trials, it has demonstrated potential for early assessment of response to immunotherapy in metastatic melanoma patients. In a prospective trial of 15 treatment-naïve metastatic melanoma patients, Lau et al reported tumor regression and significant alterations in tumor vascularity after 12 weeks of immunotherapy [17]. However, they also found that DWI and heterogeneity metrics, including apparent diffusional kurtosis, were unable to distinguish between true progression and pseudoprogression. This underscores the potential differences in tumor evolution and dynamics between animal models and humans. In a clinical study of melanoma brain metastases, lesions exhibiting pseudoprogression showed significantly lower median PV at the 90th percentile compared to true progression [45]. This becomes particularly evident when considering the rapid tumor growth observed in this study. Our findings are consistent with prior studies that emphasize the limitations of DWI- and DCE-MRI in capturing immune-mediated alterations in the tumor microenvironment during this early treatment interval [31], further underscoring the need for advanced imaging techniques to monitor immunotherapy response.

Immunotherapy has transformed the treatment landscape of melanoma and improved the five-year overall survival rates to over 50% in patients with advanced disease. However, not all patients respond to immunotherapy, and most fail to achieve durable outcomes [46]. Primary and acquired resistance mechanisms remain incompletely understood but include inadequate T-cell tumor infiltration, immunosuppressive tumor microenvironment factors, alternative immune checkpoint expression, JAK1/2 mutations disrupting interferon-gamma signaling, impaired antigen presentation, and neoantigen loss [47–49]. In clinical oncology, size-based criteria like iRECIST are used to evaluate response. However, consistent with previous studies, our findings showed no significant difference in tumor volume between experimental groups five days after treatment, highlighting the need for alternative non-invasive biomarkers to monitor early tumor microenvironment dynamics and detect immunotherapy response when conventional size-based measures remain inconclusive [30, 50].

Our findings have potential implications for the timing and interpretation of imaging studies, demonstrating that the tumor microenvironment displays anti-tumoral changes as early as five days after therapy. While early imaging biomarkers of immunotherapy response are critical for guiding treatment decisions, particularly given the variable response rates to immunotherapy, additional and especially later follow-up timepoints may yield valuable insights, given the stability of quantitative MR values in our study. While quantitative mpMRI promises to provide valuable insights into the tumor microenvironment and dynamics [25, 28], its ability to capture the complex, oftentimes non-linear biological changes over time induced by immunotherapy may be limited and explain why quantitative mpMRI has not entered the clinical arena [51–53]. Nonetheless, with further advancements in MRI and sequence developments such as “IMPULSED” to assess mean cell size, early response assessment may become feasible [30].

Our study has several limitations that should be considered when interpreting the results. First, the sample size was relatively small, underscoring the need for larger cohorts to confirm and generalize our findings. Due to the exploratory study design, significance levels were not adjusted for multiple testing, which, however, would not affect the significance of the difference in ADC between groups at follow-up (*p* = 0.001) or the increase in tumor volume observed in both groups (both *p* ≤ 0.004). Second, a clinical 3-T scanner was used with limited spatial resolution compared to dedicated small animal scanners. This may have influenced the accuracy of perfusion measurements, which depend on assessment of the small mouse abdominal aorta (< 1 mm). However, our findings may therefore be more reflective of true clinical capabilities and constraints, enhancing the translational relevance of our work. Third, the experimental design and observation period were restricted by the rapid tumor growth observed, particularly following twelve days after tumor inoculation, preventing longer observational periods. With accelerated tumor evolution and murine response in this experimental study, clinical translation is restricted. Also, a control group was used to assess nonresponse, while true nonresponse following immunotherapy may show different patterns. Fourth, the F16-B10 murine melanoma model, while well-established and standardized, exhibited variability in observed growth patterns as shown by our data. This variability reflects the inherent microscopic and macroscopic heterogeneity of melanoma. To mitigate segmentation bias and address potential central necrosis, a volumetric analysis and two reader consensus readings were employed. Finally, tumor biology in animals and humans may diverge substantially following immunotherapy, with potential impact on the concept and timing of early response assessment, as well as the evaluation of therapeutic efficacy.

In conclusion, we report the efficacy of a combined intraperitoneal anti-PD-L1 and anti-CTLA-4 immunotherapy in a F16-B10 murine melanoma model by DWI MRI and *ex vivo* fluorescence immunohistochemistry. Our findings highlight the complex interplay between immune infiltration, dynamic changes in the tumor microenvironment, and quantitative imaging findings following immunotherapy. The immunotherapy group displayed significantly lower ADC values five days after therapy compared to the control group, most likely reflecting the increased immune infiltration by CD8+ lymphocytes. In this small cohort, the mono-exponential IVIM model and DCE-MRI did not yield quantitative imaging parameters capable of distinguishing between experimental groups at follow-up. These findings underscore the importance of developing robust, multiparametric imaging strategies to accurately assess early immunotherapy response and guide clinical decision-making in oncology.

## Supplementary information

ELECTRONIC SUPPLEMENTARY MATERIAL

## Data Availability

The data that support the findings of this study are available on reasonable request from the corresponding author.

## Abbreviations

ADC: Apparent diffusion coefficient
*D*: Free water diffusion
*D**: Pseudodiffusion
DAPI: 4′,6-Diamidino-2-phenylindole
DCE-MRI: Dynamic contrast-enhanced MRI
DWI: Diffusion-weighted imaging
*f*: Perfusion fraction
IVIM: Intravoxel incoherent motion
mpMRI: Multiparametric MRI
MRI: Magnetic resonance imaging
PF: Plasma flow
PV: Plasma volume
TILs: Tumor infiltrating lymphocytes
TUNEL: Terminal deoxynucleotidyl transferase deoxyuridine triphosphate (dUTP) nick-end labeling
