# Supplementary material for: Quantitative response assessment of combined immunotherapy in a murine melanoma model using multiparametric MRI
Source: Eur Radiol Exp. 2025 Jun 14;9:59. doi: 10.1186/s41747-025-00597-8 (PMC12167185; doi:10.1186/s41747-025-00597-8)
Supplement: Supplementary file 1 — ELECTRONIC SUPPLEMENTARY MATERIAL [file 41747_2025_597_MOESM1_ESM.pdf]

# Quantitative response assessment of combined immunotherapy in a murine melanoma model using multiparametric MRI

## ELECTRONIC SUPPLEMENTARY MATERIAL

**MRI sequence protocol**

1. T1w GRE sequence

Imaging plane: axial

Repetition Time (TR): 44 ms

Echo Time (TE): 3.46 ms

Flip Angle: 15°

Field of View (FOV): 150 × 103 $mm^2$

Slice Thickness and Gap: 0.5 mm, no gap

Matrix Size: 416 × 286 (96 slices)

Resolution: 0.36 × 0.36 × 0.5 $mm^3$

Fat Suppression: none

Acquisition Time: 4:10 min

Acceleration Factor: 2 (GRAPPA)

2. Diffusion-Weighted Imaging (DWI)

Pulse sequence: Diffusion-weighted single-shot spin-echo EPI sequence

Imaging plane: Axial

Repetition Time (TR): 3200 ms

Echo Time (TE): 52.0 ms

b-values: 0, 200, 800 s/mm$^2$

Diffusion Directions: 6, monopolar

Field of View (FOV): 86 × 86 mm$^2$

Slice Thickness and Gap: 2.0 mm, 0.2 mm gap

Matrix Size: 128 × 128 (10 slices)

Spatial Resolution: 0.67 × 0.67 × 2.2 mm$^3$

Echo Planar Imaging (EPI) Factor: 64 (single-shot)

Signal Averaging: 8 (×1), 4 (×6), 4 (×6) averages for b values 0, 200, 800 s/mm$^2$

Fat Suppression: SPAIR

Acquisition Time: 3:18 min

Acceleration factor: 4 (GRAPPA)

3. Intravoxel Incoherent Motion (IVIM)

Pulse sequence: Diffusion-weighted single-shot spin-echo EPI sequence

Imaging plane: Axial

Repetition Time (TR): 3200 ms

Echo Time (TE): 52.0 ms

b-values: 0, 10, 30, 50, 80, 120, 200, 500, 800 s/mm$^2$

Field of View (FOV): 86 × 86 mm$^2$

Slice Thickness and Gap: 2.0 mm, 0.2 mm gap

Matrix Size: 128 × 128 (10 slices)

Spatial Resolution: 0.67 × 0.67 × 2.2 mm$^3$

Echo Planar Imaging (EPI) Factor: 64 (single-shot)

Signal Averaging: 8 (×1) and 4 (×6) averages for b values 0 and all others, respectively

Fat Suppression: SPAIR

Acquisition Time: 10:59 min

Acceleration factor: 4 (GRAPPA)

Fitting Model: tri-exponential (with fixed free water diffusion coefficient Dwater = 3.0× 10-3 mm2/s).

4. Dynamic Contrast-Enhanced (DCE) MRI

Pulse sequence: echo-sharing 3D gradient-echo sequence (TWIST)

Imaging plane: Axial

Repetition Time (TR): 5.50 ms

Echo Time (TE): 1.67 ms

Temporal Resolution: 1.85 s per volume (300 measurements)

Flip Angle: 19°

Field of View (FOV): 100 × 100 $mm^2$

Slice Thickness and Gap: 1.5 mm, no gap

Matrix Size: 128 × 128 (24 slices)

Spatial Resolution: 0.78 × 0.78 × 1.5 $mm^3$

Fat Suppression: None

Acquisition Time: 9:23 min

Acceleration factor: 2 (GRAPPA)
